# Supplementary material for: A reappraisal of the Middle Triassic chirotheriid Chirotherium ibericus Navás, 1906 (Iberian Range NE Spain), with comments on the Triassic tetrapod track biochronology of the Iberian Peninsula
Source: PeerJ. 2015 Jun 23;3:e1044. doi: 10.7717/peerj.1044 (PMC4485737; doi:10.7717/peerj.1044)
Supplement: Table S1 [file peerj-03-1044-s001.doc]

| Icnotaxon | Age and/or facies | Locality | Reference |
| --- | --- | --- | --- |
| *Dicynodontipus* isp. | Anisian, Buntsandstein | Montseny-Llobregat (Barcelona) | Valdiserri et al. (2009);  Fortuny et al. (2012) |
| *Procolophonichnium* isp. | Triassic *sensu lato* | Cueto Cucón (Cantabria) | Demathieu & Saiz de Omeñaca (1990) |
| *Procolophonichnium* isp. | Anisian, Buntsandstein | Montseny-Llobregat (Barcelona) | Valdiserri et al. (2009) |
| *Rhynchosauroides santanderensis* | Triassic *sensu lato* | Puentenansa (Cantrabria) | Demathieu & Saiz de Omeñaca (1976, 1977) |
| *Rhynchosauroides virgiliae* | Buntsandstein | Rillo de Gallo (Guadalajara) | Demathieu et al. (1978) |
| *Rhynchosauroides* isp. | Buntsandstein | Río Arandilla (Guadalajara) | Demathieu et al. (1978) |
| *Rhynchosauroides extraneus* | Trissic *sensu lato* | Puentesana (Cantabria) | Demathieu & Saiz de Omeñaca (1979) |
| *Rhynchosauroides simulans* | Triassic *sensu lato* | Puentesana (Cantabria) | Demathieu & Saiz de Omeñaca (1979) |
| *Rhynchosauroides* isp. | Anisiense (Fortuny et al., 2012), Buntsandstein | Tagamanent (Barcelona) | Calzada (1987) |
| *Rhynchosauroides* cf. *beasleyei* | Anisiense (Fortuny et al., 2012), Buntsandstein | Tagamanent (Barcelona) | Calzada (1987) |
| *Rhynchosauroides* isp. | Triásico sensu lato | Cueto Cucón (Cantabria) | Demathieu & Saiz de Omeñaca (1990) |
| *Rhynchosauroide*s isp. | Buntsandstein | Peñarroyas (Teruel) | Ezquerra et al. (1995) |
| *Rhynchosauroides* isp. | Anisian, Buntsandstein | Montseny-Llobregat (Barcelona) | Valdiserri et al. (2009), Fortuny et al. (2012) |
| *Rhynchosauroides* isp. | Olenekian – Anisian, Buntsandstein | Desierto de Las Palmas (Castellón) | Gand et al. (2010) |
| *Rhynchosauroides* isp. | Anisian, Buntsandstein | Corbalán (Teruel) | Gand et al. (2010) |
| *Rhynchosauroides* isp. | Anisian, Buntsandstein | Moncayo (Zaragoza) | In this work |
| *Rotodactylus* isp. | Anisian, Buntsandstein | Montseny-Llobregat (Barcelona) | Valdiserri et al. (2009), Fortuny et al. (2012) |
| *Batrachopus* isp. | Buntsandstein | Rillo de Gallo (Guadalajara) | Demathieu et al. (1978) |
| *Batrachopus* isp. | Muschelkalk | Riba de Santiuste (Guadalajara) | Demathieu et al. (1978) |
| *Brachychirotherium* cf. *gallicum* | Keuper | Cambil (Jaén) | Pérez-López (1993) |
| *Brachychirotherium gallicum* | Anisian, Muschelkalk | Boniches (Cuenca) | Gand et al. (2010) |
| *Brachychirotherium* isp. | Anisian, Muschelkalk | Boniches (Cuenca) | Gand et al. (2010) |
| Chirotheroiid (*Chirotherium gallicum*; *Cheirotherium*) | Buntsandstein | Rillo de Gallo (Guadalajara) | Díaz-Martínez & Pérez-García, 2012 (Leonardi, 1959; Calderón, 1897) |
| *Chirtotherium barthii (Chirotherium ibericus*; *Chirosaurs ibericus*) | Anisian (in this work), Buntsandstein | Moncayo (Zaragoza) | In this work, (Leonardi, 1959; Navas, 1906) |
| *Chirtotherium barthii* (*Chirotherium* isp.) | Buntsandestein | San Gaudioso del Moncayo (Zaragoza) | Leonardi (1959) (Gómez de Llarena, 1917) |
| *Chirotherium* isp. | Muschelkalk | Boniches (Cuenca) | Lapparent et al. (1965) |
| *Chirotherium* isp. | Buntsandstein | Aragoncillo (Guadalajara) | Demathieu et al. (1978) |
| *Nomen dubium* (*Chirotherium catalaunicum*) | Buntsandstein | Cervelló (Barcelona) | Fortuny et al. (2011) (Casanovas et al., 1979) |
| *Hyloidichnus (Chirotherium* sp.) | Late Permian (Gand et al., 2010) | Delia y Soler (Mallorca) | Gand et al. (2010) (Calafat et al., 1986-1987) |
| *Chirotheium barthii* | Anisian (Fortuny et al., 2012) | Tagamanent (Barcelona) | Calzada (1987) |
| *Chirotherium i*sp. | Muschelkalk | Nuévalos (Zaragoza) | García-Bartual et al. (1996) |
| *Chirotherium barthii* | Anisian, Buntsandstein | Montseny-Llobregat (Barcelona) | Valdiserri et al. (2009), Fortuny et al. (2012) |
| *Chirotherium barthii* | Anisian, Buntsandstein | Corbalán (Teruel) | Gand et al. (2010) |
| *Chirotherium* isp. | Anisian, Muschelkalk | Corbalán  (Teruel) | Gand et al. (2010) |
| *Isochirotherium* isp. | Buntsandstein | Ermita de la Virgen del Buen Labrado (Guadalajara) | Demathieu et al. (1978) |
| *Isochirotherium soergeli* | Anisian, Buntsandstein | Montseny-Llobregat (Barcelona) | Valdiserri et al. (2009), Fortuny et al. (2012) |
| *Isochirotherium* cf. *coureli* | Anisian, Buntsandstein | Corbalán (Teruel) | Gand et al. (2010) |
| *Synaptichnium* isp. | Buntsandstein | Rillo de Gallo (Guadalajara) | Demathieu et al. (1978) |
| *Synaptichnium* isp. | Muschelkalk | Riba de Santiuste (Guadalajara) | Demathieu et al. (1978) |
| *Synaptichnium* isp. | Anisian (Fortuny et al., 2012) | Tagamanent (Barcelona) | Calzada (1987) |
| *Synaptichnium* isp. | Buntsandstein | Peñarroyas (Teruel) | Ezquerra et al. (1995) |
| *Synaptichnium* sp. | Anisian, Buntsandstein | Montseny-Llobregat (Barcelona) | Valdiserri et al. (2009), Fortuny et al. (2012) |
| *Chirotheriid* | (Middle) Ladinian-early Carnian, Muschelkalk | Catalonian Basin (Barcelona) | Fortuny et al. (2012) |
| *Chirotheriid* | Anisian, Buntsandstein | Moncayo (Zaragoza) | In this work |
| “Group” formed by the Chirotherium-Isochirotherium-Brachychirotherium ichnogenera | Ladinian, Muschelkalk | Paredes de Sigüenza (Guadalajara) | Meléndez &Moratalla (2014) |
| Grupo Dinosauroide | Triassic *sensu lato* | Puentenansa (Cantabria) | Demathieu & Saiz de Omeñaca (1976, 1977) |
| *Coelurosaurichnus* sp. | Buntsandstein | Aragoncillo (Guadalajara) | Demathieu et al. (1978) |
| *Eubrontes* sp. | Rhaetian | Carrascosa de Arriba (Soria) | Pascual-Arribas & Latorre-Macarrón (2000) |
| *Anchisauripus* sp. | Rhaetian | Carrascosa de Arriba (Soria) | Pascual-Arribas & Latorre-Macarrón (2000) |
| *Coelurosaurichnus perriauxi* | Anisian, Muschelkalk | Boniches (Cuenca) | Gand et al. (2010) |
| *Paratrisauropus latus* | Anisian, Muschelkalk | Boniches (Cuenca) | Gand et al. (2010) |
| Tipo 2 | Triassic *sensu lato* | Puentenansa (Cantrabria) | Demathieu & Saiz de Omeñaca (1976, 1977) |
| Tipo 4 | Triassic *sensu lato* | Puentenansa (Cantrabria) | Demathieu & Saiz de Omeñaca (1976, 1977) |
| Tipo 1 | Triassic *sensu lato* | Puentenansa (Cantabria) | Demathieu & Sainz de Omeñaca (1979) |
| Tipo 2 | Triassic *sensu lato* | Puentenansa (Cantabria) | Demathieu & Sainz de Omeñaca (1979) |
| Tipo 3 | Triassic *sensu lato* | Puentenansa (Cantabria) | Demathieu & Sainz de Omeñaca (1979) |
| Tipo 4 | Triassic *sensu lato* | Puentenansa (Cantabria) | Demathieu & Sainz de Omeñaca (1979) |
| Tipo B | Buntsandstein | Delia and Soler (Mallorca) | Calafat et al. (1986-1987) |
| Tipo Ca | Buntsandstein | Delia and Soler (Mallorca) | Calafat et al. (1986-1987) |
| Tipo Cb | Buntsandstein | Delia and Soler (Mallorca) | Calafat et al. (1986-1987) |
| Tipo D | Buntsandstein | Delia and Soler (Mallorca) | Calafat et al. (1986-1987) |
| Archosaur | Landian, Buntsandstein | Santisteban del Puerto (Jaén) | Demathieu et al. (1999) |
| Unnamed Morphotype | Anisian, Buntsandstein | Moncayo (Zaragoza) | In this work |

Supplementary information Table S1: Summary of all the Iberian Triassic tracks published in the Iberian Peninsula.

REFERENCES

Calafat F, Fornós JJ, Marzo M, Ramos-Guerrero E, Rodríguez-Perea A. 1986–1987. Icnología de vertebrados de la facies Buntsandstein de Mallorca. *Acta Geológica Hispánica* 21-22:515-520.

Calderón S. 1897. Una huella de *Cheirotherium* de Molina de Aragón. *Actas de la Sociedad Española de Historia Natural* 26:27-29.

Calzada S. 1987. Niveles fosilíferos de la facies Buntsandstein (Trías) en el sector norte de los Catalánides. *Cuadernos de Geología Ibérica* 11:115-130.

Casanovas Cladellas ML, Santafé Llopis JV, Gómez Alba J. 1979. Presencia de *Chirotherium* en el Triásico Catalán. *Boletín Informativo del Instituto Provincial de Paleontologia de Sabadell* 9:34-42.

Demathieu G, Saiz de Omeñaca J. 1976. La faunei chnologique du Trias de Puentenansadans son environnement paleogeographique (Santander, Espagne). *Bulletin de la Société Géologique de France* 18:1251-1256.

Demathieu G, Saiz de Omeñaca J. 1977. Estudio del *Rhynchosauroides santanderiensis*, n. sp., y otras nuevas huellas de pisadas en el Trias de Santander, con notas sobre el ambiente paleográfico. *Acta geológica hispánica* 12(1): 49-54.

Demathieu G, Saiz de Omeñaca J. 1979. Características y significado del *Rhynchosauroides extraneus* n. sp., *Rh*. *simulans* n. sp. y otras nuevas huellas del Triásico de Cantabria. *Boletín de la Real Sociedad Española de Historia Natural. Sección geológica* 77(1): 91-99.

Demathieu G, Saiz de Omeñaca J. 1990. Primeros resultados del estudio de un nuevo yacimiento de icnofauna triásica en Peña Sagra (Cantabria. España). *Estudios Geológicos* 46(1-2): 147-150.

Demathieu G, Ramos A, Sopeña A. 1978. Fauna icnológica del Triásico del extremo noroccidental de la Cordillera Ibérica (Prov. de Guadalajara).*Estudios Geológicos* 34:175-186.

Demathieu GR, Pérez‐López A, Pérez‐Lorente F. 1999. Enigmatic ichnites in the middle Triassic of Southern Spain. *Ichnos* 6(4):229-237.

Díaz-Martínez I, Pérez-García A. 2012. Historical and comparative study of the first Spanish vertebrate paleoichnological record and bibliographic review of the Spanish chiroteroiid footprints. *Ichnos* 19:141-149.

Ezquerra R, Zurita C, Soria AR, Martínez P. 1995. Icnitas de vertebrados en las facies Buntsandstein (Triásico inferior) del Macizo de Montalbán (Peñarroyas, Provincia de Teruel). *Geogaceta* 18:109-112.

Fortuny J, Bolet A, Sellés AG, Cartanyà J, Galobart À. 2011. New insights on the Permian and Triassic vertebrates from the Iberian Peninsula with emphasis on the Pyrenean and Catalonian basins. *Journal of Iberian Geology* 37:65-86.

Fortuny J, Bolet A, Oms O, Bonet M, Diviu M, Rodríguez P, Galobart À. 2012. Permian and Triassic ichnites from the Catalonian and Pyrenean basins (Eastern Iberian Peninsula).State of the art and new findings. *¡Fundamental!* 20:73-75.

Gand G, De La Horra R, Galán-Abellán B, López-Gómez J, Barrenechea JF, Arché A, Benito MI. 2010. New ichnites from the Middle Triassic of the Iberian Ranges (Spain): Paleoenvironmental and paleogeographical implications. *Historical Biology* 22(1):40-56.

García-Bartual M, Rincón R, Hernándo S. 1996. Propuesta de una nueva técnica de estudio mediante análisis digital de imagen en huellas quiroteroides encontradas en el Triásico de Nuévalos (Provincia de Zaragoza). *Cuadernos de Geología Ibérica* 20:301-312.

Gómez de Llarena J. 1917. La estratigrafía del Moncayo. *Boletín de la Real Sociedad Española de Historia Natural* 17:568-572.

Lapparent AF de, Le Joncour M, Mathieu A, Plus B 1965. Découverte en Espagne d´empreintes de pas de Reptiles mésozöıques. *Boletín de la Real Sociedad Española de Historia Natural (Geología)* 63: 225-230.

Leonardi P. 1959.Orme chirotheriane triassich espagnole. *Estudios Geológicos* 15:235-245.

Meléndez Hevia N, Moratalla Garcia J. 2014. Los Arroturos: new reptile tracksite from the Muschelkalk (Middle Triassic) of Paredes de Sigüenza (Guadalajara province, Spain). 74th annual meeting Society of vertebrate paleontology, Abstracts Book, Berlin, 186.

Navás L. 1906. El *Chirosaurus ibericus* sp. nov. *Boletín de la Sociedad Aragonesa de Ciencias Naturales* 5:208-213.

Pascual-Arribas C, Latorre-Macarrón P. 2000. Huellas de *Eubrontes* y *Anchisauripus* en Carrascosa de Arriba (Soria, España). *Boletín geológico y minero* 111(1):21-32.

Pérez-López A. 1993. Estudio de las huellas de reptil, del icnogénero *Brachychirotherium*, encontradas en el Triásico subbetico de Cambil (Jaén).

*Estudios Geológicos* 49:77-83.

Valdiserri D, Fortuny J, Galobart A. 2009. New insight on old material: Triassic tetrapods footprints in Catalonia (NE Iberian Peninsula). Tenth International Symposium on Mesozoic Ecosystems, Abstract book, Teruel, 163–164.
